# Supplementary material for: Deletion of wfs1 Impairs Oligodendrocyte Precursor Cells Dorsal Distribution and Myelination Through the wfs1-hmgcs1 Axis in Zebrafish
Source: Biology (Basel). 2026 Aug 16;15(16):1402. doi: 10.3390/biology15161402 (PMC13510151; doi:10.3390/biology15161402)
Supplement: Supplementary file 1 [file biology-15-01402-s001.zip › File S1-Transcriptome sequencing data and differentially expressed genes (DEGs) in wfs1-- zebrafish at 5 dpf/2.KEGG/wfs1KOvsWT/UP/src/dre04217.html]

dre04217


- dre:768248

- Up regulated genes

768248(6.07490717007993)

- dre:561021

- Up regulated genes

561021(2.14284154145004)

- dre:337413

- Up regulated genes

337413\_1(9.49663367060307)

- dre:110438871

- Up regulated genes

110438871(1.82412391238383)

- dre:100149436

- Up regulated genes

100149436(1.04343414699703)

- dre:393444

- Up regulated genes

393444(1.27911287872533)

- dre:566185

- Up regulated genes

566185(6.72123660882883)

- dre:561187

- Up regulated genes

100148591(1.46970663726292)

- dre:336426

- Up regulated genes

336426(1.55368624578632)

- dre:393186

- Up regulated genes

393186\_1(3.79579966743607)

Close
